# Therapeutic prospects of ceRNAs in COVID-19

**DOI:** 10.3389/fcimb.2022.998748

**Published:** 2022-09-20

**Authors:** Lin Liu, Yao Zhang, Yu Chen, Yueshui Zhao, Jing Shen, Xu Wu, Mingxing Li, Meijuan Chen, Xiaobing Li, Yuhong Sun, Li Gu, Wanping Li, Fang Wang, Lei Yao, Zhuo Zhang, Zhangang Xiao, Fukuan Du

**Affiliations:** ^1^ Laboratory of Molecular Pharmacology, Department of Pharmacology, School of Pharmacy, Southwest Medical University, Luzhou, China; ^2^ Cell Therapy & Cell Drugs of Luzhou Key Laboratory, Luzhou Science and Technology Bureau, Luzhou, China; ^3^ South Sichuan Institute of Translational Medicine, Luzhou, China; ^4^ Experiment Medicine Center, The Affiliated Hospital of Southwest Medical University, Luzhou, China; ^5^ Department of Oncology, Affiliated Hospital of Southwest Medical University, Luzhou, China

**Keywords:** COVID-19, lncRNA, miRNA, circRNA, ceRNA

## Abstract

Since the end of 2019, COVID-19 caused by SARS-CoV-2 has spread worldwide, and the understanding of the new coronavirus is in a preliminary stage. Currently, immunotherapy, cell therapy, antiviral therapy, and Chinese herbal medicine have been applied in the clinical treatment of the new coronavirus; however, more efficient and safe drugs to control the progress of the new coronavirus are needed. Long noncoding RNAs (lncRNAs), microRNAs (miRNAs), and circular RNAs (circRNAs) may provide new therapeutic targets for novel coronavirus treatments. The first aim of this paper is to review research progress on COVID-19 in the respiratory, immune, digestive, circulatory, urinary, reproductive, and nervous systems. The second aim is to review the body systems and potential therapeutic targets of lncRNAs, miRNAs, and circRNAs in patients with COVID-19. The current research on competing endogenous RNA (ceRNA) (lncRNA-miRNA-mRNA and circRNA-miRNA-mRNA) in SARS-CoV-2 is summarized. Finally, we predict the possible therapeutic targets of four lncRNAs, MALAT1, NEAT1, TUG1, and GAS5, in COVID-19. Importantly, the role of PTEN gene in the ceRNA network predicted by lncRNA MALAT1 and lncRNA TUG1 may help in the discovery and clinical treatment of effective drugs for COVID-19.

## 1 Introduction

In December 2019, a smoke-free war between humans and viruses occurred. COVID-19 is an infectious disease caused by SARS-CoV-2, which invades human cells through the ACE2 (angiotensin-converting enzyme 2) receptor (Atzrodt et al., 2020; Li et al., 2020a); it enters the body similarly to SARS-CoV and its clinical manifestations are similar to those of SARS-CoV (Shang et al., 2020; Huang et al., 2020b). SARS-CoV-2 uses the respiratory tract as the main invasion site to cause acute respiratory diseases with fever, cough, and shortness of breath as the main symptoms. Studies have shown that in addition to respiratory system symptoms, SARS-CoV-2 infection can also cause symptoms in the digestive, nervous, cardiovascular, reproductive, immune, and urinary systems (Chowdhury et al., 2020; Hassanein et al., 2020; Li et al., 2020a; Wang et al., 2021b). SARS-CoV-2 affects many systems in the human body, eventually leading to multiple organ failure (Majumder and Minko, 2021). This disease has brought a huge challenge to humans, and antiviral drugs discovered thus far are limited to delaying the clinical progress of COVID-19 (Stasi et al., 2020). The main mechanism by which COVID-19 causes human pathogenesis and its treatment require further study. Understanding the pathogenesis of COVID-19 and suitable treatment targets will help effective clinical treatment.

Studies have shown that analyzing the ceRNA network established by lncRNA/circRNA-miRNA-mRNA in SARS-CoV-2 infection is promising for the development of effective treatment methods (Arora et al., 2020). LncRNAs are RNA molecules with more than 200 nucleotides (Moazzam-Jazi et al., 2021), that participate in the antiviral immune response of host cells (Henzinger et al., 2020; Moazzam-Jazi et al., 2021). CircRNA is a circulating non-coding RNA (Wu et al., 2021) that plays an important role in gene regulation; however, the virus-encoded circRNA requires further study (Huang et al., 2019). Through the GO and KEGG enrichment analysis of the clinical manifestations in COVID-19 patients by Wu et al., we learned that lncRNA and circRNA also play a role in many diseases. Moreover, lncRNAs and circRNAs also play a role in organ damage, indicating that lncRNAs and circRNAs have an impact on the body systems of COVID-19 patients (Wu et al., 2021). MiRNA is a non-coding single-stranded RNA of approximately 19-28 nucleotides in length that can regulate gene expression and protein synthesis at the transformation level (Abedi et al., 2021). It can combine with the complementary sequence of the viral RNA chain to form a silent complex, which can destroy the viral RNA (Henzinger et al., 2020). MiRNAs may be one of the reasons why men are more severely infected with the new coronavirus than women ( Pontecorvi et al., 2020).

Currently, there are no effective drugs available for the clinical treatment of COVID-19. We aimed to determine whether lncRNAs, circRNAs, and miRNAs could provide relevant targets for COVID-19 treatment. In this article, we review the research progress on lncRNAs, miRNAs, and circRNAs in various body systems during COVID-19 infection. Most importantly, we review the current role of lncRNAs, miRNAs, and circRNAs in COVID-19 and describe the potential mechanism of the ceRNA network in the course of COVID-19 infection for the discovery of potential therapeutic targets for COVID-19 and provide a new direction for the treatment of COVID-19.

## 2 The impact of COVID-19 on body systems

**Figure 1:**
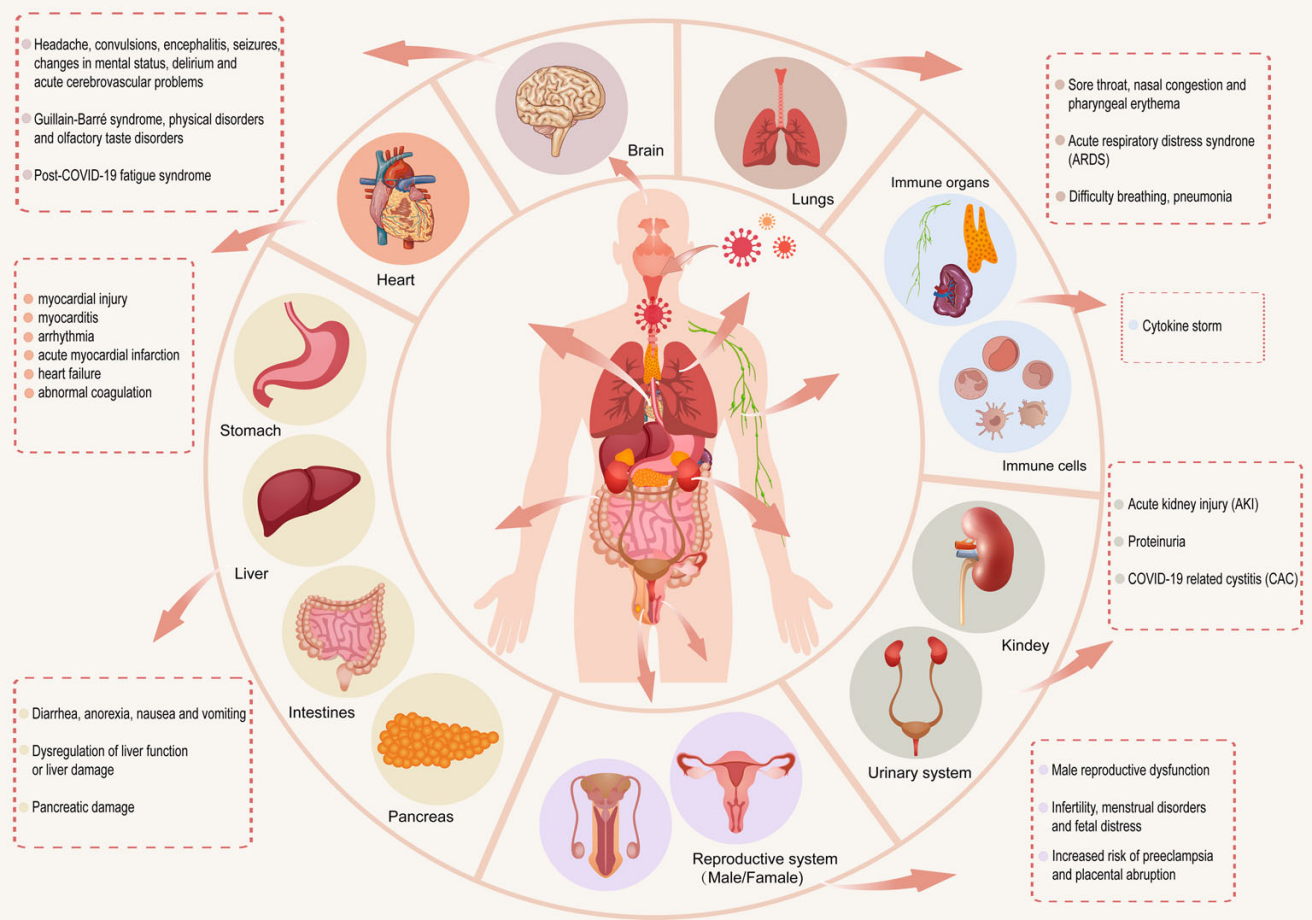
After SARS-CoV-2 enters the human body, it has different effects on the respiratory, immune, digestive, circulatory, urinary, reproductive and nervous systems of the human body.

SARS-CoV-2 enters the human body through the ACE2 receptor (Li et al., 2020a) and exerts different effects on various systems of the human body. In this section, we review the effects of coronavirus on the respiratory, immune, digestive, circulatory, urinary, reproductive, and nervous systems after viral entry (**Figure 1**).

### 2.1 Respiratory system

SARS-CoV-2 directly affects the respiratory system, including the nose, throat, trachea, bronchi, and lungs. ACE2 is expressed in cells of the nose and oropharynx and is conducive to the entry of the virus into the human body (Kaya et al., 2021). Studies have shown that nitric oxide (NO) produced by the paranasal sinuses can diffuse into the lungs and bronchi. NO inhalation can be used as a treatment method to improve the symptoms of ARDS caused by SARS-CoV-2 infection. However, the antiviral effect of NO on COVID-19 patients’ needs further study (Martel et al., 2020). A retrospective study showed that a sore throat is the most common otolaryngological symptom in COVID-19 patients, and nasal congestion and pharyngeal erythema can lead to complications in COVID-19 patients (El-Anwar et al., 2020). The SARS-CoV-2 receptor ACE2 and cofactor TMPRSS2 are expressed in the lungs, trachea, and bronchus. Tyrosine-protein kinase receptor UFO can promote SARS-CoV-2 entry into the human body and infect pulmonary and bronchial epithelial cells, which can cause breathing difficulties, pneumonia, and acute respiratory distress syndrome (ARDS). These conditions are more likely to occur in severely ill patients (Lukassen et al., 2020) (Cinesi Gomez et al., 2020; Satarker and Nampoothiri, 2020; Sato et al., 2021; Wang et al., 2021c). A retrospective study of the autopsy results of COVID-19 patients showed that 100% of the 113 patients died of ARDS, which is the main cause of death in COVID-19 patients (Eketunde et al., 2020). The nose and oropharynx, as gateways for SARS-CoV-2 entry, are important research targets for preventing the spread of the virus. ARDS is one of the complications that leads to the death of COVID-19 patients. Inhaled NO has been clinically effective in the treatment for ARDS, but the anti-SARS-CoV-2 effect of NO requires further research.

### 2.2 Immune system

The immune system is divided into immune cells and immune organs. There is an interactive relationship between the immune system and SARS-CoV-2 infection. The immune system plays an important role in preventing diseases and protecting the body from viruses, bacteria, tumors, and other invaders (Chowdhury et al., 2020). During SARS-CoV-2 infection, immune cells in the body undergo immune metabolism reprogramming, which is inseparable from inflammation. With an increase in viral load, SARS-CoV-2 causes a cytokine storm by activating monocytes, macrophages, dendritic cells, T cells, mast cells, and neutrophils, leading to the activation and secretion of IL-6 and other inflammatory cytokines (Kempuraj et al., 2020; Khadke et al., 2020). Some patients suddenly deteriorate in the later stage of the disease or during the recovery process, which may be related to the cytokine storm produced in the body of COVID-19 patients (Ye et al., 2020). The immune responses produced by patients at different stages of infection are different. The intensity of a patient’s immune response determines the phenotype (asymptomatic, mild, moderate, or severe) of SARS-CoV-2 infected patients (Kumar, 2021). Currently, antiviral drugs, immunomodulatory treatment methods, and convalescent plasma are the main methods used to treat cytokine storms caused by SARS-CoV-2 infection (Copaescu et al., 2020; Santa Cruz et al., 2021).

### 2.3 Digestive system

COVID-19 is closely associated with the digestive system. Studies have shown that organs expressing ACE2 in the digestive system are at risk of being infected by SARS-CoV-2, such as the gastrointestinal tract, liver, and pancreas. The main gastrointestinal symptoms of COVID-19 patients are diarrhea, anorexia, nausea, and vomiting (Galanopoulos et al., 2020; Jin et al., 2020; Lin et al., 2020). Studies have shown that SARS-CoV-2 infection can directly cause liver damage (Wang et al., 2020d), and SARS-CoV-2 may also cause liver dysfunction or damage through the overexpression of ACE2 receptors in gallbladder cells (Patel et al., 2020; Mohamed et al., 2021). Patients with a normal liver have mild-to-moderate liver damage after infection with SARS-CoV-2, and patients with cirrhosis have an increased risk of death after infection (Cabibbo et al., 2021). Patients with severe COVID-19 have higher serum alanine aminotransferase (ALT) and aspartate aminotransferase (AST) levels than non-severe patients, which may be related to immune-mediated systemic inflammation in patients with COVID-19 (Mohamed et al., 2021). The expression of ACE2 in the pancreas is higher than that in the lungs (Liu et al., 2020a). COVID-19 patients with pancreatic injury are more severely affected on admission to hospital. In a study of 52 patients with coronavirus infection, the incidence of pancreatic injury was 17% (Wang et al., 2020a). In addition, ACE2 is also expressed in pancreatic islets and pancreatic exocrine glands, which has an impact on insulin production and can trigger blood sugar elevation (Liu et al., 2020a; Somasundaram et al., 2020).

The gastrointestinal tract may be a potential route for SARS-CoV-2 transmission (Lin et al., 2020). The severity of hepatic and pancreatic damage is proportional to the severity of COVID-19 infection. Therefore, the liver and pancreas of patients with severe COVID-19 should be carefully monitored during treatment.

### 2.4 Circulatory system

The circulatory system includes the cardiovascular and lymphatic systems. The high expression of ACE2 receptors in the heart is similar to that in the lungs, which makes the heart one of the main targets of the virus (Wicik et al., 2020; Singh et al., 2021). Cardiovascular complications in COVID-19 patients include myocardial injury, myocarditis, arrhythmia, acute myocardial infarction, heart failure, and coagulation abnormalities (Kwenandar et al., 2020; Long et al., 2020). A study of 416 COVID-19 patients in Wuhan, China showed that heart damage was related to higher hospital mortality rate (19.7%) of COVID-19 patients (Shi et al., 2020). Studies have shown that COVID-19 patients with cardiovascular risk factors (such as high blood pressure) have a higher mortality rate, which may be related to increased inflammation in SARS-CoV-2 infection or the use of angiotensin-converting enzyme inhibitors (ACEIs) or angiotensin II receptor blockers (ARBs) that inhibit the renin-angiotensin-aldosterone system (RAAS) and cause a compensatory increase in ACE2 (Guzik et al., 2020; Azevedo et al., 2021). The impact of COVID-19 on the lymphatic system is mainly reflected in the nervous system (Wostyn, 2021), and its impact on the circulatory system requires further exploration.

### 2.5 Urinary system

The urinary system includes the kidneys, ureters, bladder, and the urethra. The impact of SARS-CoV-2 on the urinary system mainly manifests in the kidneys. The kidneys of mild and moderate patients show subclinical renal abnormalities, and acute kidney injury (AKI) is more likely to occur in critically ill patients (Martinez-Rojas et al., 2020). Dysregulation of the immune response driven by SARS-CoV-2 may be one of the causes of AKI (Ahmadian et al., 2021). Studies have shown that AKI is the most common extrapulmonary complication of COVID-19 (Yang et al., 2020). According to kidney biopsy data, in addition to AKI, proteinuria is also a common sign (Akilesh et al., 2021). Studies have also shown that infection with SARS-CoV-2 can cause COVID-19-related cystitis (CAC), which is related to increased levels of pro-inflammatory cytokines in the patient’s urine (Dhar et al., 2020; Lamb et al., 2020). The urinary system exhibits different clinical symptoms under the influence of immune cells; however, the specific mechanism requires further study.

### 2.6 Reproductive system

Regarding the impact of COVID-19 on the reproductive system, ACE2 is highly expressed in the testis of the male reproductive system (Sharma et al., 2021). A study of scRNA-seq data in adult human testes showed that male gonads may be infected with SARS-CoV-2, which may cause male reproductive dysfunction (Wang and Xu, 2020). The increase in pro-inflammatory cells and decrease in androgens in men infected with SARS-CoV-2 may lead to a decreased gonadal function (Dutta and Sengupta, 2021).

SARS-CoV-2 may also infect the ovaries, uterus, vagina, and placenta in the female reproductive system through the expression of ACE2 receptors, leading to infertility, menstrual disorders, and fetal distress (Jing et al., 2020). Pregnant women infected with SARS-CoV-2 have a lower incidence of vertical transmission of the virus to newborns, which may be related to the lower expression of ACE2 and TMPRSS2 in the placenta and an increase in SARS-CoV-2 specific antibodies and IgG during pregnancy (Atyeo et al., 2021; Wastnedge et al., 2021); however, it may cause inflammation of the placenta, which increases the risk of pre-eclampsia and placental abruption during pregnancy (Hosier et al., 2020).

ACE2 is expressed at a high level in testicular tissues compared to that in the ovaries and uterus. Therefore, male patients may be slightly more affected by SARS-CoV-2 infection than females (Li et al., 2020a).

### 2.7 Nervous system

The nervous system can be divided into the central and peripheral nervous systems. An analysis of the tissue-specific expression of ACE2 showed that the nervous system and lungs have similar ACE2 expressions (Wicik et al., 2020). SARS-CoV-2 can spread from the respiratory tract to the central nervous system (CNS). In the nervous system, the main manifestations include headaches, convulsions, changes in mental status, and encephalitis. Some patients experience seizures and delirium. Critically ill COVID-19 patients are more likely to have acute cerebrovascular problems (Asadi-Pooya and Simani, 2020; Ferrarese et al., 2020; Flores-Silva et al., 2021; Singh et al., 2021).

A retrospective study showed that the peripheral nerve complications of COVID-19 patients mainly manifest as Guillain-Barré syndrome (GBS), physical disorders, and smell and taste which are the earliest manifestations of SARS-CoV-2 infection of nerves and can be used as diagnostic markers (Ellul et al., 2020; Karuppan et al., 2021). GBS is affected by COVID-19, which is likely due to poor immune system regulation caused by COVID-19 (Rajdev et al., 2020).

In addition, studies have shown that COVID-19 patients suffer from post-COVID-19 fatigue syndrome, which mainly manifests as chronic fatigue, sleep disturbance, cognitive impairment, muscle pain, and depressive symptoms (Bornstein et al., 2021; Mackay, 2021; Wostyn, 2021). These symptoms are related to an increase in resistance to cerebrospinal fluid (CSF) outflow, which leads to congestion of the lymphatic system and promotes the accumulation of toxic substances in the nervous system (Wostyn, 2021).

SARS-CoV-2 can enter the brain *via* the olfactory and cervical lymphatic pathways (Bostanciklioglu, 2020), and its effects on the central and peripheral nervous systems manifest as different symptoms. Some neurologically affected COVID-19 patients have post-COVID-19 fatigue syndrome, and further research is needed to prevent this complication.

In summary, SARS-CoV-2 enters the host cells mainly through the ACE2 receptor. ACE2 is expressed in many body organs (Ni et al., 2020), such as the lungs, heart, gastrointestinal tract, liver, pancreas, kidneys, male and female gonads, and nerve tissue, which affect the organs of different body systems. The immune system influences the respiratory and gastrointestinal tract through immune regulation (Mohamed et al., 2021). Dysregulation of the immune response due to the activation of some cytokines by SARS-CoV-2 is the cause of AKI, myocardial inflammation, neuroinflammation, and GBS (Kempuraj et al., 2020; Rajdev et al., 2020; Liu et al., 2020b; Ahmadian et al., 2021).The increase in inflammatory factors caused by SARS-CoV-2 is related to male infertility, and infection of the placenta can cause placental inflammation (Hosier et al., 2020; Dutta and Sengupta, 2021). Organization of the human body is related to immune regulation and inflammatory cytokines. Targeting immunoregulatory factors may provide clinical treatment methods for COVID-19.

## 3 Research progress of lncRNA, miRNA, and circRNA in body systems of persons infected with COVID-19

### 3.1 lncRNA

Long non-coding RNA act as regulators of viral infection, and the imbalance of lncRNA expression and interaction has a certain impact on the progression of viral infection (Turjya et al., 2020).

Some lncRNAs were found to be upregulated in SARS-CoV-2 infection, such as lung adenocarcinoma transcript 1 (MALAT1) and nuclear-enriched autosomal transcript 1 (NEAT1), which were upregulated in bronchial epithelial cells (NHBE) infected with SARS-CoV-2 (Vishnubalaji et al., 2020). The upregulation of lncRNA CTB-36O1.7 activates microglia, and innate immunity plays an important role in the pathogenesis of neurological manifestations of COVID-19 (Gagliardi et al., 2021). SARS-CoV-2 invades nerves and causes the sequelae of COVID-19. LncRNAs can monitor cholinergic signaling in response to COVID-19; lncRNA DANCR and lncRNA NEAT1 affect nerve tissues through cholinergic mediators, and their upregulation may change the inflammatory state of neurons (Meydan et al., 2020). Upregulation of lncRNA MALAT1 may also have anti-inflammatory effects in acute kidney injury (Paniri and Akhavan-Niaki, 2020). In a study on SARS-CoV-2 infection of respiratory epithelial cells, the expression of LASI, TOSL, and NEAT1 lncRNAs was upregulated in patients with high VL, while the expression of MALAT1 was not changed (Devadoss et al., 2021).

In SARS-CoV-2 infection, there is also a downregulation of related lncRNA expression. Studies have shown that lncRNA GAS5 is downregulated in lipopolysaccharide-treated pulmonary epithelial cells, which is a marker of ARDS (Li et al., 2018). Cytokine storm is one of the contributing factors for the development of ARDS; lncRNA NORAD, lncRNA RAD51-AS1, and lncRNA lnrCXCR4 are lncRNAs that target important cytokines in the COVID-19 cytokine storm, and their downregulation may also downregulate the expression of their target cytokines, improve pro-inflammatory immunity, and reduce the cytokine storm in the process (Morenikeji et al., 2020).

In addition, studies have shown that the silencing of MALAT1 can reduce the inflammatory response in lung injury, and the silencing of MALAT1 expression may reduce the prevalence of cytokine storms in SARS-CoV-2 patients (Henzinger et al., 2020; Vishnubalaji et al., 2020).

ACE2 is an important receptor responsible for S protein binding that mediates SARS-CoV-2 entry (Arora et al., 2020). Infertile men have higher levels of ACE2 expression in the testes are more likely to be infected with SARS-CoV-2 than men with normal fertility (Shen et al., 2020). Simultaneously, it can be inferred that men may experience infertility after SARS-CoV-2 infection. Nine of these testis-specific lncRNAs, including GRM7-AS3, ARHGAP26-AS1, BSN-AS1, KRBOX1-AS1, CACNA1C-IT3, AC012361.1, FGF14-IT1, AC012494.1, and GS1-24F4.2, revealed that SARS-CoV-2 infection in infertile men is a diagnostic possibility (Sabetian et al., 2021).

### 3.2 miRNA

MicroRNAs play an important role in antiviral defense by stimulating the release of inflammatory cytokines, thereby changing the response of cells to viral infections and playing the most significant role in lung and heart diseases (Guterres et al., 2020). Here, we review the roles of miRNAs in the respiratory, circulatory, immune, digestive, urinary, reproductive, and nervous systems of patients with COVID-19, and provide a reference for the treatment of COVID-19.

#### 3.2.1 Respiratory system

The role of MiR-200c-3p, miR-1307-3p, miR-22, and miR-146a in the respiratory system deserves attention. Studies have shown that miR-200c-3p is the main regulatory target of ACE2 in cells of the respiratory system; miR-200c-3p is highly expressed in ARDS, and has a negative correlation with ACE2. Inhibition of miR-200c-3p can promote ACE2 expression and prevent ARDS lung injury (Li et al., 2018; Bozgeyik, 2021). A study on the prediction of miRNAs in the SARS-CoV-2 genome revealed that the problems of hypoxia and increased risk of lung infection in SARS-CoV-2 infected persons were related to miR-1307-3p (Arisan et al., 2020). MiR-22 and miR-146a cause SARS-CoV-2 infection, emphysema, and shortness of breath complications (Yuka and Yilmaz, 2021). Inhibiting the expression of miR-200c-3p and regulating the expression of miR-1307-3p, miR-22, and miR-146a play a significant role in preventing and reducing the risk of infection complications in COVID-19 patients.

#### 3.2.2 Circulatory system

MiRNAs play a role in the control of cardiomyocytes and in the treatment of heart disease (Wojciechowska et al., 2017). Studies have shown that miR-200c can regulate the expression of ACE2 in cardiomyocytes *in vitro*, and its overexpression can inhibit ACE2 in cardiomyocytes (Lu et al., 2020a). Therefore, the overexpression of miR-200c could reduce the risk of heart infection with SARS-CoV-2. Cardiovascular fibrosis is an important cause of heart failure (HF), and is enhanced during inflammation. MiR-122 can play a regulatory role in cardiovascular inflammation and fibrosis. Upregulation of ACE2 promotes increases in atrial natriuretic peptide and brain natriuretic peptide levels, increasing the likelihood of cardiovascular fibrosis and HF exacerbation (Xu et al., 2020a). Overexpression of miR-122 regulates the SIRT6-ELA-ACE2 signaling pathway, exacerbating cardiovascular fibrosis (Liu et al., 2020c). However, ACE2 acts as a receptor for SARS-CoV-2, suggesting that inhibition of miR-122 may inhibit the progression of cardiac fibrosis, attenuate the inflammatory response, and prevent the development of HF in COVID-19 patients. Studies have shown that COVID-19 patients with diabetes have an increased risk of HF. Overexpression of miR-133a can protect against cardiac fibrosis and impaired contraction, and prevent further production of HF (Mishra et al., 2020). Studies have shown that miR-21, miR-155, miR-208a, and miR-499 in COVID-19 patients were upregulated compared to healthy controls and can be used as a predictor of chronic myocardial injury and inflammation. Compared to healthy subjects, miR-126 expression in COVID-19 patients is downregulated (Garg et al., 2021) and miR-126 can play a role in vascular endothelial function and peripheral angiogenesis in patients with type 2 diabetes (Lin et al., 2017; Zampetaki et al., 2010).

In conclusion, inhibiting miR-200c, miR-122, and miR-133a expression can reduce the risk of cardiovascular fibrosis and HF. MiR-21, miR-155, miR-208a, and miR-499 are markers for predicting myocardial inflammation and injury in COVID-19 patients. The role of miR-126 downregulation in the cardiovascular system requires further investigation.

#### 3.2.3 Immune system

MiRNAs mediate host immunity by regulating immune cells (Mishra et al., 2020. Some miRNAs are involved in immune regulation, some of which have antiviral effects. Additionally, miRNA-targeted host genes of SARS-CoV-2 are involved in viral pathogenesis (Karimi et al., 2021). Studies have found that host miRNAs play antiviral roles during SARS-CoV-2 infection, such as miR-17-5p, miR-20b-5p, and miR-323a-5p, has-miR-17-5p, has-miR-20b-5p, hsa-miR323a-5p (Khan et al., 2020; Abu-Izneid et al., 2021). However, overexpression of human miR-1207-5p, a regulator of viral spike protein, may enhance inflammatory responses in COVID-19 patients (Bertolazzi et al., 2020). Rahaman et al. targeted host genes through predicted 34 SARS-CoV-2 miRNAs to facilitate the proliferation of the virus in the host (Rahaman et al., 2021). Therefore, miRNAs that target viruses may be effective in reducing viral proliferation.

#### 3.2.4 Digestive system

According to a review by Farshad Abedi et al., the predicted MD2-5p, MR359-5p, and MR345-5p virus-encoded miRNAs can activate FOXO3, ADIPOQ, ADIPOR1, and ADAR genes, which may damage the liver of COVID-19 patients (Abedi et al., 2021). Liver miR-122 is associated with 28-day ICU mortality in COVID-19 patients (Gutmann et al., 2022). There are relatively few studies on miRNAs in the digestive system of COVID-19 patients, and further research is needed.

#### 3.2.5 Urinary system

Studies have shown that miR-18 and miR-125b upregulate ACE2 expression in nephropathy, which may provide a potential therapeutic option for the treatment of COVID-19-related nephropathy (Widiasta et al., 2020). Acute kidney injury (AKI) and COVID-19-associated cystitis (CAC) are complications of the urinary system in COVID-19 patients (Dhar et al., 2020; Yang et al., 2020), and their relationship with miRNAs in COVID-19 patients’ needs to be further explored.

#### 3.2.6 Reproductive system

MiR-125a-5p, miR-125b-5p, miR-574-5p, and miR-936 are ACE2 regulators related to male infertility, which is helpful in investigating male infertility after SARS-CoV-2 infection (Sabetian et al., 2021). Investigating the three miRNAs that bind directly to SARS-CoV-2 (miR-21b, miR-29c, and miR-98) or indirectly affect SARS-CoV-2 replication and immunity (miR-146a, miR-150, and miR-155), researchers found that the expression in pregnant women infected with SARS-CoV-2 (SIPW) was upregulated relative to expression in uninfected pregnant women (UPW). In addition, the increase in miR-146 in SIPW helped to control severe COVID-19 (Saulle et al., 2021). However, the specific mechanism in COVID-19 patients’ needs further study.

#### 3.2.7 Nervous system

SARS-CoV-2 spike gene triggers inflammation of the nervous system by releasing exosomes loaded with miR-148a and miR-590 (Mishra and Banerjea, 2021). Hsa-miR-146a-5p, hsa-miR-124-3p, hsa-miR-20a-5p, and hsa-miR-145-5p have prominent roles in neurological disease (Wang et al., 2020b); among them, miR-146a-5p could be a potential target for COVID-19 therapy (Tang et al., 2020a). Moreover, hsa-miR-124-3p can regulate SARS-CoV-2 infection (Prasad et al., 2021), hsa-miR-20a-5p is a host gene with antiviral activity (Sardar et al., 2020), and hsa-miR-145-5p is a potential marker that distinguishes severe and asymptomatic COVID-19 patients (Parray et al., 2021). These four miRNAs regulate the nervous system of COVID-19 patients, and further research is needed.

### 3.3 circRNA

CircRNAs play an important regulatory role in viral infections and as host anti-virals. According to data from SARS-CoV-2 infected Calu-3 cells, host circRNA was abundantly expressed in human lung epithelial cells and has a potential regulatory effect in SARS-CoV-2 infection. Hsa_circ_0080941, hsa_circ_0080942, and hsa_circ_0067985 expressions were upregulated and targeted the corresponding miRNAs by indirectly upregulating the expression of mRNA; hsa_circ_0005630, hsa_circ_0001681, and hsa_circ_0060927 are circRNAs that were found to be downregulated in the study and indirectly downregulated mRNA expression by targeting the corresponding miRNAs; circRNA plays a role in SARS-CoV-2 infection through indirect regulation of gene expression (Yang et al., 2021a). At present, there is limited research on circRNAs in COVID-19, and further research is required.

The current research progress on lncRNAs, miRNAs, and circRNAs in various systems of COVID-19 patients is summarized in **Table 1**.

**Table 1:** Relationship between lncRNA, miRNA, circRNA and various systems in COVID-19 patients.

ncRNA	Part/System	Expression	References
lncRNA MALAT1	bronchial epithelial cells	up	(Vishnubalaji et al., 2020)
lncRNA NEAT1	bronchial epithelial cells	up	(Vishnubalaji et al., 2020)
lncRNA CTB-36O1.7	nervous system	up	(Gagliardi et al., 2021)
lncRNA DANCR	nervous system	up	(Meydan et al., 2020)
lncRNA NEAT1	nervous system	up	(Meydan et al., 2020)
lncRNA MALAT1	kidney	up	(Paniri and Akhavan-Niaki, 2020)
lncRNA LASI	airway epithelial cells	up	(Devadoss et al., 2021)
lncRNA TOSL	airway epithelial cells	up	(Devadoss et al., 2021)
lncRNA NEAT1	airway epithelial cells	up	(Devadoss et al., 2021)
lncRNA GAS5	lung	down	(Li et al., 2018)
lncRNA NORAD	immune system	down	(Morenikeji et al., 2020)
lncRNA RAD51-AS1	immune system	down	(Morenikeji et al., 2020)
lncRNA lnrCXCR4	immune system	down	(Morenikeji et al., 2020)
lncRNA MALAT1	immune system	silence	(Vishnubalaji et al., 2020), (Henzinger et al., 2020)
lncRNA GRM7-AS3	testis	/	(Sabetian et al., 2021)
lncRNA ARHGAP26-AS1	testis	/	(Sabetian et al., 2021)
lncRNA BSN-AS1	testis	/	(Sabetian et al., 2021)
lncRNA KRBOX1-AS1	testis	/	(Sabetian et al., 2021)
lncRNA CACNA1C-IT3	testis	/	(Sabetian et al., 2021)
lncRNA AC012361.1	testis	/	(Sabetian et al., 2021)
lncRNA FGF14-IT1	testis	/	(Sabetian et al., 2021)
lncRNA AC012494.1	testis	/	(Sabetian et al., 2021)
lncRNA GS1-24F4.2	testis	/	(Sabetian et al., 2021)
miR-200c-3p	lung	/	(Bozgeyik, 2021) (Li et al., 2018)
miR-1307-3p	respiratory system	/	(Arisan et al., 2020)
miR-200c	cardiomyocytes	/	(Lu et al., 2020a)
miR-122	heart	/	(Liu et al., 2020c)
miR-133a	heart	/	(Mishra et al., 2020)
miR-21	heart	up	(Garg et al., 2021)
miR-155	heart	up	(Garg et al., 2021)
miR-208a	heart	up	(Garg et al., 2021)
miR-499	heart	up	(Garg et al., 2021)
miR-126	heart	down	(Garg et al., 2021)
miR-17-5p	immune system	/	(Khan et al., 2020)
miR-20b-5p	immune system	/	(Khan et al., 2020)
miR-323a-5p	immune system	/	(Khan et al., 2020)
miR-1207-5p	immune system	/	(Bertolazzi et al., 2020)
MD2-5p	liver	/	(Abedi et al., 2021)
MR359-5p	liver	/	(Abedi et al., 2021)
MR345-5p	liver	/	(Abedi et al., 2021)
miR-18	kidney	/	(Widiasta et al., 2020)
miR-125b	kidney	/	(Widiasta et al., 2020)
miR-125a-5p	male reproductive system	/	(Sabetian et al., 2021)
miR-125b-5p	male reproductive system	/	(Sabetian et al., 2021)
miR-574-5p	male reproductive system	/	(Sabetian et al., 2021)
miR-936	male reproductive system	/	(Sabetian et al., 2021)
miR-21b	female reproductive system	up	(Saulle et al., 2021)
miR-29c	female reproductive system	up	(Saulle et al., 2021)
miR-98	female reproductive system	up	(Saulle et al., 2021)
miR-146a	female reproductive system	up	(Saulle et al., 2021)
miR-150	female reproductive system	up	(Saulle et al., 2021)
miR-155	female reproductive system	up	(Saulle et al., 2021)
miR-148a	nervous system	/	(Mishra and Banerjea, 2021)
miR-590	nervous system	/	(Mishra and Banerjea, 2021)
hsa_circ_0080941	immune system	out of balance	(Yang et al., 2021a)
hsa_circ_0080942	immune system	out of balance	(Yang et al., 2021a)
hsa_circ_0067985	immune system	out of balance	(Yang et al., 2021a)
hsa_circ_0005630	immune system	out of balance	(Yang et al., 2021a)
hsa_circ_0001681	immune system	out of balance	(Yang et al., 2021a)
hsa_circ_0060927	immune system	out of balance	(Yang et al., 2021a)

## 4 The role of ceRNA in COVID-19

Viral RNAs can affect host miRNAs and thus the corresponding host mRNAs through ceRNA networks (Yuka and Yilmaz, 2021). Perturbation of ceRNA networks may have an impact on disease, explain disease processes, and provide opportunities for new therapies (Salmena et al., 2011). Currently, numerous studies on ceRNAs are performed in cancer, such as lncRNA/circRNA-miRNA-mRNA, which is associated with overall survival in lung adenocarcinoma ( Wu et al., 2020a; Wang et al., 2021d). The discovery of potential therapeutic targets in the ceRNA network and prognosis of patients with hepatocellular carcinoma can provide therapeutic directions (Long et al., 2019; Chen et al., 2021b). The circRNA-miRNA-mRNA ceRNA network can play different roles in promoting NPC progression of nasopharyngeal carcinoma (Li et al., 2021) and provide relevant therapeutic targets for breast cancer (Wang et al., 2021a). In this section we review current research on the role of ceRNAs in COVID-19 (**Table 2**).

**Table 2:** The impact of ceRNA in COVID-19.

lncRNA	miRNA	mRNA	Effect	References
lncRNA GAS5	miR-200c-3p	ACE2	Promote the development of ARDS.	(Li et al., 2018)
lncRNA DANCR	miR-19a-3p/ miR-335-5p	HIF1a/CCR7/TLR4	Differentiate between mild and severe patients with COVID-19.	(Meydan et al., 2020)
lncRNA NEAT1	miR-19a-3p/ miR-335-5p	HIF1a/CCR7/TLR4	Differentiate between mild and severe patients with COVID-19.	(Meydan et al., 2020)
lncRNA Gm26917	miR-124-3p	Ddx58	Possibility to reduce SARS-CoV-2 replication.	(Arora et al., 2020)
circRNA	miRNA	mRNA	Effect	References
circ_0000479	miR-149-5p	Ddx58	Possibility to reduce Hantaan Virus replication.	(Lu et al., 2020b) (Arora et al., 2020)
ssc_circ_009380	miR-22	IL-6/CCL5/Ddx58	Plays an anti-inflammatory role in TGEV.	(Nahand et al., 2020)

### 4.1 lncRNA-miRNA-mRNA

LncRNAs and miRNAs are involved in the pathogenesis of SARS-CoV-2 and the host antiviral immune defense mechanism (Henzinger et al., 2020). LncRNAs can be used as sponges of miRNAs to play a role in competitive endogenous RNA (ceRNA) (Tay et al., 2014; Turjya et al., 2020), and reduce miRNA degradation of their target mRNA (Tay et al., 2014; Arora et al., 2020). COVID-19 patients have a higher incidence of death due to ARDS. Studies have shown that downregulation of lncRNA GAS5 expression reduces the expression of ACE2 by regulating the expression of miR-200c-3p, thus preventing the occurrence of ARDS lung injury, and reducing the chance of death in COVID-19 patients caused by ARDS (Li et al., 2018). The lncRNAs DANCR and NEAT1 can act as “sponges” for miRNAs (miR-19a-3p and miR-335-5p) to target inflammation-related transcripts (TNF, IL-6, and CHRNA7) and regulate inflammation by preventing the activity of miRs (Meydan et al., 2020). In addition, studies have shown an interaction between SARS-CoV-2 M/N proteins and RIG-I (also known as Ddx58, retinoic acid-inducible gene 1). The former antagonizes the production of type I and type III interferons, whereas the latter inhibits the production of interferon β (IFN-β), providing opportunities for enhanced viral replication (Chen et al., 2020; Zheng et al., 2020; Wu et al., 2020b). According to the analysis and research of Shweta Arora et al., lncRNA Gm26917 can reduce the degradation of Ddx58 by sponging miR-124-3p (Arora et al., 2020), thus inferring that network interactions of this ceRNA have the potential to increase the chance of SARS-CoV-2 replication.

### 4.2 circRNA-miRNA-mRNA

CircRNAs can act as sponges to regulate miRNA target genes (Demirci and Sacar Demirci, 2021). The regulation of host mRNA by SARS-CoV-2 circRNA may promote viral replication, and host circRNA has a regulatory effect on the COVID—19 genes. In the study of viral infection in calu-3 cells, viral and human circRNA differentially expressed miRNAs, which have a competitive interaction with mRNA, and the function of viral circRNA could be determined (Cai et al., 2021). Demirci et al. predicted that circRNAs play the role of ceRNAs in SARS-CoV-2 infection. There are 200 human circRNAs targeted by has-miR-6891-5p, and the upregulated expression of has-miR-6891-5p may play a regulatory role in reducing ORF3a gene expression (Demirci and Sacar Demirci, 2021). The specific regulatory effect of host circRNAs on the genes of COVID-19 requires further study.

In addition, some single-stranded RNA viruses may serve as a reference for COVID-19 treatment. Hantaan virus and transmissible gastroenteritis virus (TGEV) are both single-stranded RNA viruses (Nahand et al., 2020; Singh et al., 2020), which are similar to SARS-CoV-2 (Wang et al., 2020c). Studies have shown that Ddx58 is highly upregulated in SARS-CoV-2 infected cells, thereby increasing viral replication (Arora et al., 2020). Circ_0000479 uses sponge miR-149-5p to target Ddx58 to regulate its expression in the Hantaan virus through its ceRNA function, forming the circ_0000479-miR-149-5p-RIG-I (Ddx58, retinoic acid-inducible gene 1) regulatory axis in HTNV infection (Arora et al., 2020; Lu et al., 2020b), and the role of this regulatory axis in SARS-CoV-2 infection needs to be further explored. Studies have shown that ssc_circ_009380 activates the NF-κB pathway of TGEV through the sponge action of miR-22 to cause inflammation, and miR-22 can also exert anti-inflammatory effect by targeting IL-6, CCL5, and Ddx58 (Nahand et al., 2020), suggesting that miR-22 can also exert an anti-inflammatory effect on SARS-CoV-2. There are relatively few studies on circRNA-miRNA-mRNA, and the study of the role of circRNA in the ceRNA network of other single-stranded RNA viruses has some implications for the study of COVID-19.

In summary, the lncRNA GAS5-miR-200c-3p-ACE2 reduced the incidence of ARDS through the action of ceRNA. Overexpression of miR-124-3p in the lncRNA Gm26917-miR-124-3p-Ddx58 network leads to the degradation of Ddx58 and a potential to reduce SARS-CoV-2 replication. The specific mechanism of action of lncRNA DANCR/NEAT1-miR-19a-3p/miR-335-5p-HIF1a/CCR7/TLR4 in COVID-19 patients’ needs to be further elucidated. The upregulated expression of has-miR-6891-5p not only targets human circRNA, but also is the only miRNA targeting SARS-CoV-2 ORF3a. The ORF3a gene of has-miR-6891-5p and circRNA are co-expressed and may appear to compete for has-miR-6891-5p binding. The prediction of 200 human circRNAs targeted by has-miR-6891-5p, has-miR-6891-5p and ORF3a as ceRNA mechanisms requires further investigation. In addition, circRNA-miRNA-mRNA ceRNA interactions have been slightly less studied in COVID-19 patients with present. The roles of circ_0000479-miR-149-5p-Ddx58 in HTNV and ssc_circ_009380-miR-22-Ddx58 in TGEV have implications for the study of SARS-CoV-2 therapeutic targets.

## 5 Potential therapeutic targets for COVID-19

### 5.1 Therapeutic targets related to lncRNA

IL-6 is regulated by the lncRNA NEAT1 (nuclear paraspeckle assembly transcript 1), lncRNA MALAT1 (lung adenocarcinoma transcript 1) and lncRNA Tug1 (taurine upregulated gene 1), while NLRP3 is regulated by lncRNA NEAT1 and lncRNA MALAT1. These factors affect the host immune response and play a role in controlling SARS-CoV-2 infection by blocking inflammatory cytokines.

When the body is infected with SARS-CoV-2, IL-6 and NLRP3 are the main immune components stimulated by the body’s immune response. The lncRNAs NEAT1 and MALAT1 alter host gene expression to promote antiviral response to SARS-CoV-2 infection. The function and expression of IL-6 and NLRP3 are affected by lncRNAs. Studies have shown that MALAT1 and NEAT1 can regulate IL-6 and NLRP3 inflammasomes, and the use of IL-6 and NLRP3 blockers is beneficial for reducing SARS-CoV-2’s morbidity and mortality (Paniri and Akhavan-Niaki, 2020; Laha et al., 2021). Whether IL-6 receptor blockers, such as sarilumab (NCT04315298), tocilizumab (NCT04320615, NCT04315480), and siltuximab (NCT04329650), can be used to treat COVID-19 patients is in the subject of ongoing clinical trials (Faheem et al., 2020). Additionally, studies have shown that the use of tocilizumab in most COVID-19 patients can improve their clinical symptoms (Xu et al., 2020b). An experiment on lipopolysaccharide-induced mouse ATDC5 cells showed that emodin, an anti-inflammatory drug, can block inflammatory cytokines, such as IL-6, and reduce apoptosis and inflammation by regulating the upregulation of lncRNA Tug1 expression (Yang et al., 2021b). In addition, some lncRNAs regulated by interferon-regulated (IFR) proteins STAT1 and STAT3 may provide new therapeutic targets against SARS-CoV-2 infection (Laha et al., 2021).

### 5.2 Therapeutic targets related to miRNA

#### 5.2.1 ACE2

ACE2 is an important receptor for SARS-CoV-2 infection. Inhibition of miRNA-145 expression can prevent SARS-CoV-2 from entering cells through the ACE2 receptor. Some miRNAs can act on ACE2 to regulate the cytokine storm and lung injury; in addition, miR-200c inhibits ACE2 expression to control cardiovascular complications in COVID-19 patients. In addition, miRNAs related to the regulation of immunity (antimiR-18 and antimiR-125b) play a role in the treatment of kidney disease by inhibiting ACE2.

ADAM17 can control the expression of ACE2, and applying antagomirs to miRNA-145 can increase the expression of ADAM17 and prevent SARS-CoV-2 from entering the cell (Mirzaei et al., 2021). Studies have shown that miR-302c-5p affects ACE2, which may play a role in the cytokine storm during SARS-CoV-2 infection, and has-miR-16-5p can play a role in the regulation of the IL-1β, IL-6, TNF-α, and NF-κB mTOR pathways. Both miRNAs have the potential to treat cytokine storms caused by SARS-CoV-2 infection (Wicik et al., 2020). Hsa-miRNA 200b-3p, hsa-miRNA 200c-3p, and miRNA-429 directly target the ACE2 receptor and regulate the key proteins required for viral entry into host lung epithelial cells (Chauhan et al., 2021). MiR-200c can inhibit the expression of ACE2 receptors in cardiomyocytes and has a certain effect on preventing cardiovascular complications in patients with COVID-19 (Mirzaei et al., 2021). According to Arghiani et al., the use of miRNAs that regulate immunity to inhibit ACE2 has therapeutic potential for the treatment of COVID-19 nephropathy (Arghiani et al., 2021). Studies have shown that antimiR-18 and antimiR-125b can attenuate the effects of ACE2 and have an effect on COVID-19-related nephropathy (Widiasta et al., 2020).

#### 5.2.2 IL-6

A number of studies have shown that IL-6 is a new target for the treatment of COVID-19, and IL-6 inhibitors have the potential to treat COVID-19 (Coomes and Haghbayan, 2020; Gubernatorova et al., 2020) (Tang et al., 2020b; Xu et al., 2020b). Studies have shown that miR-146a-5p could be used as a potential target for COVID-19 treatment (Tang et al., 2020a). Studies showed a decrease in miR-146a-5p expression and an increase in IL-6 levels in patients with COVID-19 compared to that in healthy people. Patients with COVID-19 pneumonia treated with the anti-IL-6 receptor tocilizumab had better outcomes than those who did not respond to this treatment (Sabbatinelli et al., 2021). A retrospective study of 146 COVID-19 patients showed that IL-6 receptor blocking tocilizumab is beneficial for reducing the mortality rate of severe COVID-19 patients (Galvan-Roman et al., 2021).

#### 5.2.3 ORF1a and ORF3a

Some miRNAs can act on SARS-CoV-2 genes (ORF1ab and ORF3a), thereby affecting the proliferation of the 2019-nCoV. Several genes are targeted by human miRNAs in SARS-CoV-2. ORF1ab and ORF3a are targets of hsa-miR-203b-3p (Sacar Demirci and Adan, 2020). Studies have shown that hsa-let-7c-5p, hsa-miR-342-5p, hsa-miR-432-5p, hsa-miR-98-5p, and hsa-miR-17-5p can target ORF1ab to play a role in SARS-CoV-2 (Sacar Demirci and Adan, 2020; Banaganapalli et al., 2021), which may have an effect on the increase in SARS-CoV-2; however, the specific mechanism requires further study. In addition, the upregulated hsa-miR-6891-5p targets ORF3a and reduces its expression of ORF3a, which plays a vital role in host resistance to SARS-CoV-2 infection (Demirci and Sacar Demirci, 2021).

In addition, studies have shown that attenuating miRNAs (miR-21, miR-125b, miR-199a, miR-211, miR-138, miR-211, miR-146a, and miR-146b) involved in the inflammatory response can reduce the cytokine storm and acute lung injury in patients with COVID-19 (Abedi et al., 2021); furthermore, ARDS-induced lung injury is reduced by eliminating miR-155 (Mirzaei et al., 2021). MiR-122 is a miRNA specifically expressed in the liver, and inhibiting the expression of miR-122 has an effect on the treatment of HCV (Jopling, 2012). Some miRNAs play an important role in MERS-CoV (miRNA 628-5p, miRNA 18a-3p, and miRNA 332-3p). Studies have suggested that these miRNAs may be potential treatment targets for COVID-19 (Chauhan et al., 2021).

At present, more research on ACE2 and IL-6 as therapeutic targets related to miRNA in SARS-CoV-2 infection is available, while research on ORF1ab, ORF3a, and miRNA is currently limited; thus, further research is needed.

### 5.3 Therapeutic targets related to circRNA

CircRNAs are stable and function in untranslated viral regions. Research by Pfafenrot et al. showed that antisense(AS)_1-75 circRNA can effectively reduce viral proliferation by affecting the 5’ untranslated region of SARS-CoV-2 and plays a role in the prevention of SARS-CoV-2 infection and antiviral therapy (Pfafenrot et al., 2021). This may be a new research direction in the field of COVID-19 treatment.

CircRNAs have a high degree of stability and play an important regulatory role in viral infection and host antiviral response (Yang et al., 2021a). Studies have shown that an artificial circRNA sponge sequestered miRNA-122, thereby making the drug Miravirsen work at lower titers in HCV (hepatitis C virus) patients (Tan and Lim, 2021). At the same time, circFNDC3B and circCNOT1 can be used as potential targets for treating MERS-CoV infection (Zhang et al., 2020).

CircRNA also plays an important role in other diseases and is currently an important therapeutic target in diabetes, cardiovascular disease, neurological disease, and cancer (Huang et al., 2020a; Cai et al., 2021), Studies have shown that circRNAs can be used as therapeutic targets for viral infections (Tan and Lim, 2021), but the therapeutic targets for SARS-CoV-2 infection require further research.

The lncRNAs NEAT1, MALAT1, and Tug1 are lncRNA-related therapeutic targets for the treatment of COVID-19 patients. ACE2, IL-6, ORF1ab, and ORF3a are miRNA-related therapeutic targets for COVID-19 treatment. AS_1-75 circRNA has been shown to be of great significance in the study of SARS-CoV-2 infection. Antisense circRNAs provide a research direction for studying new antiviral drugs (Pfafenrot et al., 2021). Some circRNAs in HCV and MERS-CoV can also serve as therapeutic targets for SARS-CoV-2 infection. Potential therapeutic targets for COVID-19 are summarized in **Table 3**.

**Table 3:** Potential related therapeutic targets of lncRNA, miRNA and circRNA in COVID-19.

ncRNA		Target	Effect	References
lncRNA	NEAT1	IL-6/NLRP3	Regulation of host genes promotes antiviral responses to SARS-CoV-2.	(Paniri and Akhavan-Niaki, 2020) (Laha et al., 2021)
	MALAT1	IL-6/NLRP3	Regulation of host genes promotes antiviral responses to SARS-CoV-2.	(Paniri and Akhavan-Niaki, 2020) (Laha et al., 2021)
	Tug1	IL-6	Reduce apoptosis and inflammation.	(Yang et al., 2021b)
miRNA	miRNA-145	ADAM17/ACE2	Block SARS-CoV-2 from entering cells.	(Mirzaei et al., 2021)
	miR-302c-5p	ACE2	cytokine storm	(Wicik et al., 2020)
	has-miR-16-5p	IL-1β/IL-6/TNF-α/NF-κB mTOR	cytokine storm	(Wicik et al., 2020)
	hsa-miRNA 200b-3p	ACE2	A key protein required for regulating viral entry into host lung epithelial cells.	(Chauhan et al., 2021)
	hsa-miRNA 200c-3p	ACE2	A key protein required for regulating viral entry into host lung epithelial cells.	(Chauhan et al., 2021)
	miRNA-429	ACE2	A key protein required for regulating viral entry into host lung epithelial cells.	(Chauhan et al., 2021)
	miR-200c	ACE2	Prevention of cardiovascular complications.	(Mirzaei et al., 2021)
	miR-18	ACE2	Treating COVID-19 Kidney Disease.	(Widiasta et al., 2020)
	miR-125b	ACE2	Treating COVID-19 Kidney Disease.	(Widiasta et al., 2020)
	miR-146a-5p	IL-6	Potential targets for COVID-19 treatment.	(Tang et al., 2020a)
	hsa-miR-203b-3p	ORF1ab/ORF3a	May have implications for increased SARS-CoV-2.	(Sacar Demirci and Adan, 2020), (Banaganapalli et al., 2021)
	hsa-let-7c-5p	ORF1ab	May have implications for increased SARS-CoV-2.	(Sacar Demirci and Adan, 2020), (Banaganapalli et al., 2021)
	hsa-miR-342-5p	ORF1ab	May have implications for increased SARS-CoV-2.	(Sacar Demirci and Adan, 2020), (Banaganapalli et al., 2021)
	hsa-miR-432-5p	ORF1ab	May have implications for increased SARS-CoV-2.	(Sacar Demirci and Adan, 2020), (Banaganapalli et al., 2021)
	hsa-miR-98-5p	ORF1ab	May have implications for increased SARS-CoV-2.	(Sacar Demirci and Adan, 2020), (Banaganapalli et al., 2021)
	hsa-miR-17-5p	ORF1ab	May have implications for increased SARS-CoV-2.	(Sacar Demirci and Adan, 2020), (Banaganapalli et al., 2021)
	hsa-miR-6891-5p	ORF3a	Host resistance to SARS-CoV-2 infection.	(Demirci and Sacar Demirci, 2021)
	miR-21	/	Reduce cytokine storm and lung damage.	(Abedi et al., 2021)
	miR-125b	/	Reduce cytokine storm and lung damage.	(Abedi et al., 2021)
	miR-199a	/	Reduce cytokine storm and lung damage.	(Abedi et al., 2021)
	miR-211	/	Reduce cytokine storm and lung damage.	(Abedi et al., 2021)
	miR-138	/	Reduce cytokine storm and lung damage.	(Abedi et al., 2021)
	miR-146a	/	Reduce cytokine storm and lung damage.	(Abedi et al., 2021)
	miR-146b	/	Reduce cytokine storm and lung damage.	(Abedi et al., 2021)
	miR-155	/	Reduces lung damage from ARDS.	(Mirzaei et al., 2021)
	miR-122	/	Inhibition of miR-122 expression for HCV therapy.	(Jopling, 2012)
	miRNA 628-5p	/	Has a regulatory effect on MERS-CoV.	(Chauhan et al., 2021)
	miRNA 18a-3p	/	Has a regulatory effect on MERS-CoV.	(Chauhan et al., 2021)
	miRNA 332-3p	/	Has a regulatory effect on MERS-CoV.	(Chauhan et al., 2021)
circRNA	AS_1-75 circRNA	SARS-CoV-2 5' untranslated region	Prevention of SARS-CoV-2 infection and antiviral therapy.	(Pfafenrot et al., 2021)
	circFNDC3B	/	Treatment of MERS-CoV infection.	(Zhang et al., 2020)
	circCNOT1	/	Treatment of MERS-CoV infection.	(Zhang et al., 2020)

## 6 Predicting ceRNA interactions in SARS-CoV-2 infection

**Figure 2:**
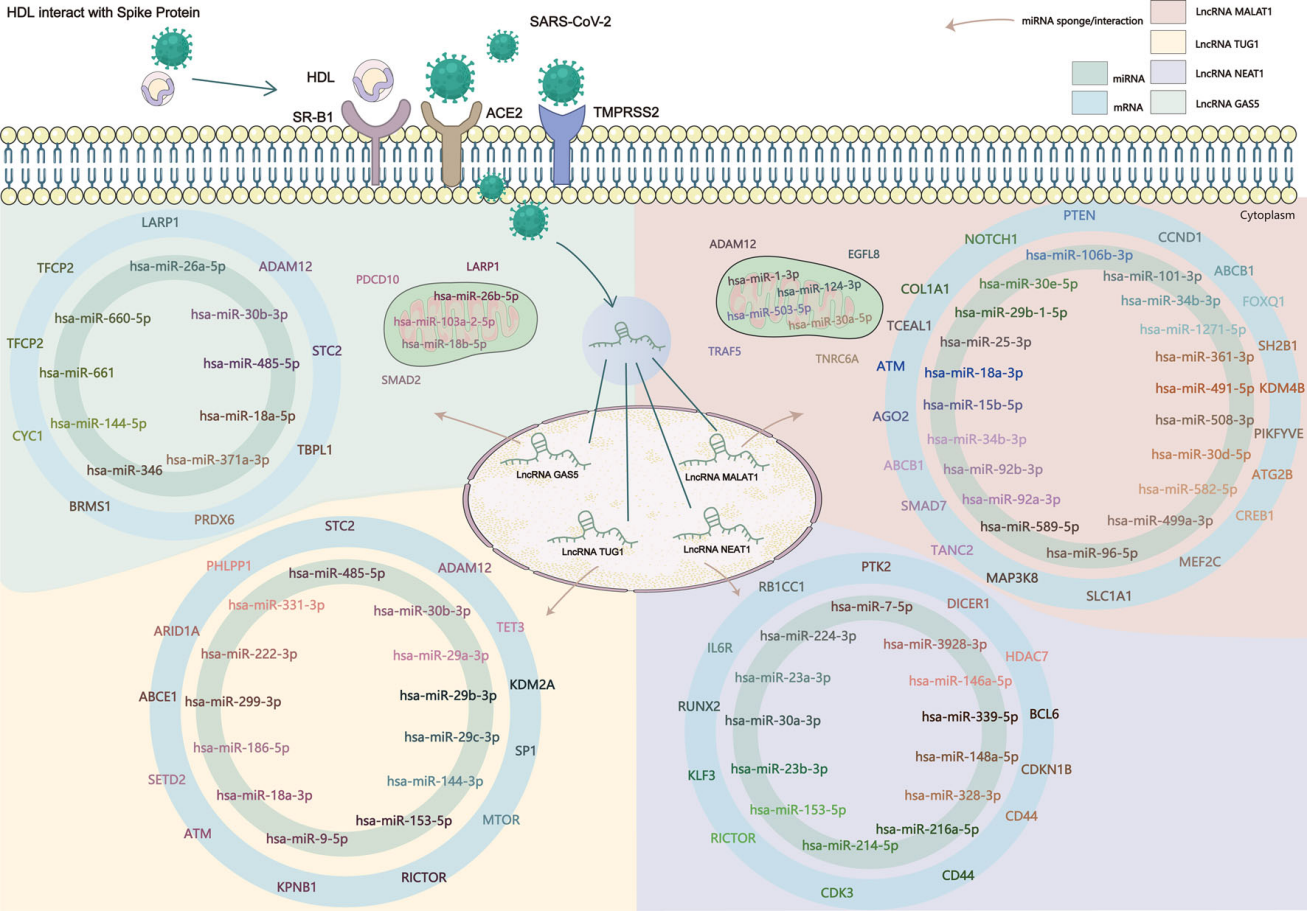
SARS-CoV-2 enters the human body through the ACE2 receptor, TMPRSS2, and SR-B1 co-receptors, affects the expression of lncRNA, and then affects the downstream gene targets through the ceRNA network constructed by lncRNA-miRNA-mRNA. The known lncRNA GAS5, lncRNA MALAT1, lncRNA NEAT1, and lncRNA TUG1 are the therapeutic targets of COVID-19, and the predicted lncRNA-miRNA-mRNA network may provide new targets in the clinical treatment and drug development of COVID-19.

**Figure 3:**
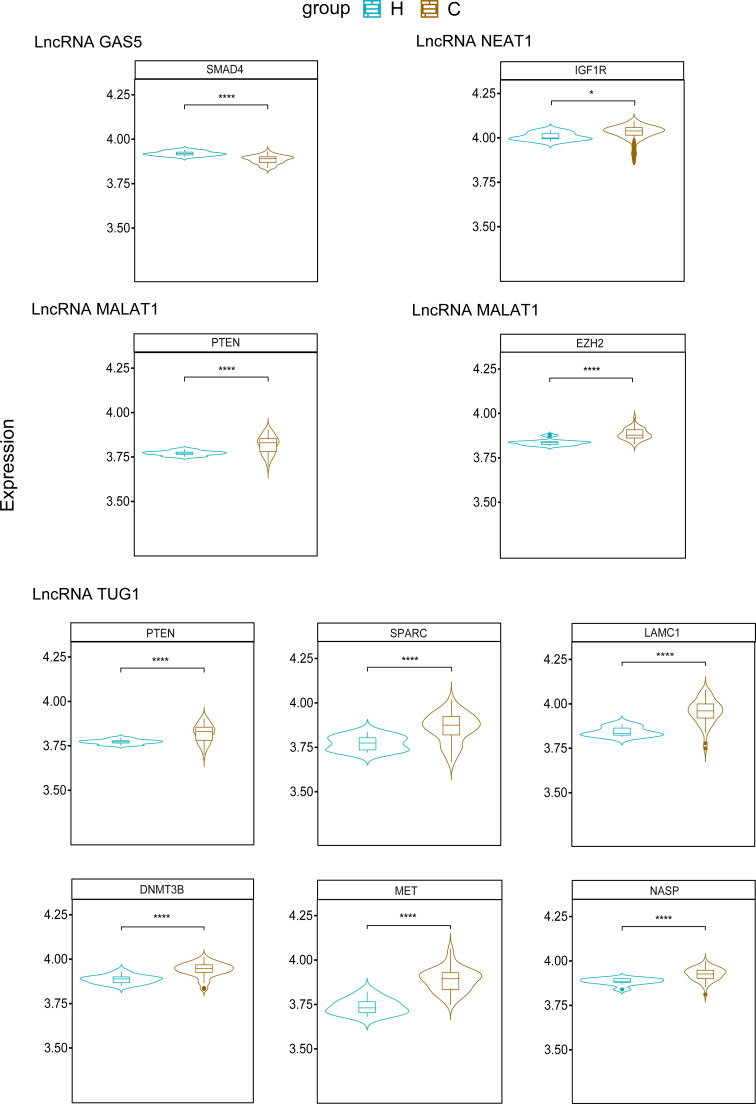
Predicted mRNAs expression of SMAD4, IGF1R, PTEN, EZH2, SPARC, LAMC1, DNMT3B, MET, NASP. (H, Health; C, COVID-19) ****P<0.0001, *P<0.05.

Existing potential therapeutic targets of the new coronavirus include lncRNA MALAT1 NEAT1 TUG1 (Paniri and Akhavan-Niaki, 2020; Laha et al., 2021). The lncRNA GAS5 regulates ACE2 expression *via* miR-200c-3p and is potentially associated with the reduction of ARDS mortality in COVID-19 patients (Li et al., 2018). We used the four lncRNAs to predict disease-related lncRNA-miRNA-mRNA ceRNA network data using LncACTdb 2.0, (http://www.bio-bigdata.net/LncACTdb2.0/, lncRNA-related ceRNA network database) and found 60 related miRNAs and 635 mRNAs. Cytoscape software was used to perform ceRNA network analysis (**Supplementary Figure**). We also downloaded the mRNA expression levels of COVID-19 patients and healthy individuals from the GEO database (GSE171110) and performed a simple analysis of the predicted mRNA gene expression levels (**Figure 2**). miRNAs play a negative regulatory role in the ceRNA axis (Salmena et al., 2011), so we screened for mRNAs that are regulated by two or more different miRNAs, which are significantly different (****P<0.0001, *P<0.05) and consistent with lncRNA expression (**Table 4** and **Figure 3**).

**Table 4:** Predicting the role of lncRNA-miRNA-mRNA in COVID-19.

lncRNA	miRNA	mRNA
lncRNA GAS5	hsa-miR-26a-5p/hsa-miR-144-5p	SMAD4
lncRNA MALAT1	hsa-miR-124-3p/hsa-miR-30d-5p/hsa-miR-101-3p	EZH2
	hsa-miR-92b-3p/hsa-miR-106b-3p/hsa-miR-25-3p	PTEN
lncRNA NEAT1	hsa-miR-214-5p/hsa-miR-7-5p	IGF1R
lncRNA TUG1	hsa-miR-29a-3p/hsa-miR-29b-3p/hsa-miR-29c-3p	DNMT3B
	hsa-miR-29b-3p/hsa-miR-29c-3p	LAMC1
	hsa-miR-144-3p/hsa-miR-222-3p	MET
	hsa-miR-29a-3p/hsa-miR-29b-3p/hsa-miR-29c-3p	NASP
	hsa-miR-29a-3p/hsa-miR-29b-3p/hsa-miR-144-3p	PTEN
	hsa-miR-29a-3p/hsa-miR-29b-3p/hsa-miR-29c-3p	SPARC

### 6.1 LncRNA GAS5

Based on existing research, the expression of lncRNA GAS5 is downregulated in COVID-19. Some studies have shown that SMAD4 can activate ACE2 in intestinal epithelial cells (Chen et al., 2021a), and downregulating the expression of SMAD4 may reduce the risk of SARS-CoV-2 infection in intestinal epithelial cells. The mechanism of the lncRNA GAS5-hsa-miR-26a-5p/hsa-miR-144-5p-SMAD4 network requires further study.

According to previous studies, lncRNA MALAT1, lncRNA NEAT1, and lncRNA TUG1 were upregulated in COVID-19.

### 6.2 LncRNA MALAT1

EZH2 and PTEN are predicted to be upregulated. Studies have shown that upregulation of EZH2 promotes the development of esophageal squamous cell carcinoma (Qiu et al., 2020). After knocking out EZH2, the expression level of ACE2 increased, and the presence of EZH2 affected ACE2 expression (Li et al., 2020b). A study comparing COVID-19 ARDS patients with ARDS due to other causes showed that the PTEN signaling pathway is activated in COVID-19 ARDS patients (Sarma et al., 2021) and plays an antiviral role as an immunomodulator (Chen and Guo, 2017). We indicated that the predicted lncRNA MALAT1-hsa-miR-124-3p/hsa-miR-30d-5p/hsa-miR-101-3p-EZH2 network may play a role in regulating ACE2 expression, whereas the lncRNA MALAT1-hsa-miR-92b-3p/hsa-miR-106b-3p/hsa-miR-25-3p-PTEN network may play a role in the inflammatory response to COVID-19.

### 6.3 LncRNA NEAT1

IGF1R, which blocks IGF-1R attenuates lung damage and reduces the risk of death in patients with COVID-19-related ARDS (Winn, 2020). The predicted lncRNA NEAT1 -hsa-miR-214-5p/hsa-miR-7-5p-IGF1R network may reduce the risk of death from ARDS in COVID-19 patients.

### 6.4 LncRNA TUG1

DNMT3B, LAMC1, MET, NASP, PTEN, and SPARC are upregulated in COVID-19. High DNMT3B expression is associated with poor prognosis in lung cancer (Yang et al., 2014). DNMT3B levels were significantly reduced in lung epithelial cells infected with SARS-CoV-2 (Muhammad et al., 2021), the predicted lncRNA TUG1 - hsa-miR-29a-3p/hsa-miR-29b-3p/hsa-miR-29c-3p-DNMT3B network may have a role in the treatment of SARS-CoV-2-infected lung epithelial cells. PTEN is also upregulated in the ceRNA network predicted by lncRNA TUG1; thus, the lncRNA TUG1-hsa-miR-29a-3p/hsa-miR-29b-3p/hsa-miR-144-3p-PTEN network has the potential to play a role in the COVID-19 anti-inflammatory response and ARDS.

However, the roles of LAMC1, MET, NASP, and SPARC in COVID-19 have not yet been elucidated. The roles of lncRNA TUG1 and the ceRNA network of these four genes in COVID-19 are merit exploration, as they may provide new research targets for clinical treatment.

We summarized the therapeutic targets of ceRNA in COVID-19 and established an lncRNA-miRNA-mRNA network diagram through prediction (**Figure 2**), which is meaningful for the subsequent discovery of new targets and drugs for COVID-19 treatment. PTEN is an mRNA regulated by two different lncRNAs (MALAT1 and TUG1) and two or more miRNAs. Previous studies have shown that lncRNA MALAT1 can regulate IL-6 and NLRP3 inflammatory cytokines (Paniri and Akhavan-Niaki, 2020), and lncRNA TUG1 plays an important role in the anti-inflammatory response to COVID-19 by blocking IL-6 cytokines (Yang et al., 2021b). PTEN also plays an antiviral role in immune regulation (Chen and Guo, 2017). There have been no studies of these two lncRNAs and PTEN in COVID-19. predicted lncRNA MALAT1 - hsa-miR-92b-3p/hsa-miR-106b-3p/hsa-miR-25-3p - PTEN and lncRNA TUG1 - hsa-miR-29a-3p/hsa-miR-29b-3p/hsa-miR-144-3p - PTEN networks may provide new targets for COVID-19 treatment and drug development, but the specific mechanism of action requires further study.

## 7 Conclusion

In the present review, the research progress of COVID-19 patients in the respiratory, immune, digestive, circulatory, urinary, reproductive, and neurological systems, as well as lncRNA, miRNA, and circRNA in various systems of COVID-19 patients and as therapeutic targets were discussed. We focused on the role of lncRNA/circRNA-miRNA-mRNA as ceRNAs in COVID-19. CeRNA networks play different regulatory roles in the development of ARDS, inflammatory activity-related transcripts and SARS-CoV-2 replication. Four lncRNAs, MALAT1, NEAT1, TUG1 and GAS5 are predicted as potential therapeutic targets in COVID-19. However, most studies on ceRNAs have focused on tumors, whereas research on 2019-nCoV is relatively scarce. In future studies, the mechanism of lncRNA/circRNA-miRNA-mRNA as a ceRNA in COVID-19 should be further explored. Finally, there are currently few effective therapeutic drugs for COVID-19 in the clinic, and the potential therapeutic targets predicted through lncRNA-miRNA-mRNA may provide new directions for drug research and treatment of COVID-19.

## Author contributions

All authors contributed to the conception and design, writing, critical revision, and final approval of the article. LL, YaZ, and YC conceived the article; ZZ, ZX, and FD supervised the study and coordinated the writing of the manuscript. All authors have read and agreed to the published version of the manuscript.

## Funding

This work was supported by the Joint Founds of Southwest Medical University and Luzhou Government (No.2020LZXNYDJ08), Grants from the Sichuan Science and Technology Program, China (No. 2022NSFSC0783), Grants of Southwest Medical University (2021ZKMS038), Funds of talent introduction and scientific research of Southwest Medical University (No.05-00040140), the Strategic Cooperation Project for Transfer and Transformation of Scientific and Technological achievements of Southwest Medical University and Lu County Government (grant no.2019LXXNYKD-07), The Project of Science and Technology Department of Sichuan Provincial of China to L.Y. (2019JDJQ0035).

## Conflict of interest

The authors declare that the research was conducted in the absence of any commercial or financial relationships that could be construed as a potential conflict of interest.

## Publisher’s note

All claims expressed in this article are solely those of the authors and do not necessarily represent those of their affiliated organizations, or those of the publisher, the editors and the reviewers. Any product that may be evaluated in this article, or claim that may be made by its manufacturer, is not guaranteed or endorsed by the publisher.
