# Supplementary figures and images for: Therapeutic prospects of ceRNAs in COVID-19
Source: Front Cell Infect Microbiol. 2022 Sep 20;12:998748. doi: 10.3389/fcimb.2022.998748 (PMC9530275; doi:10.3389/fcimb.2022.998748)

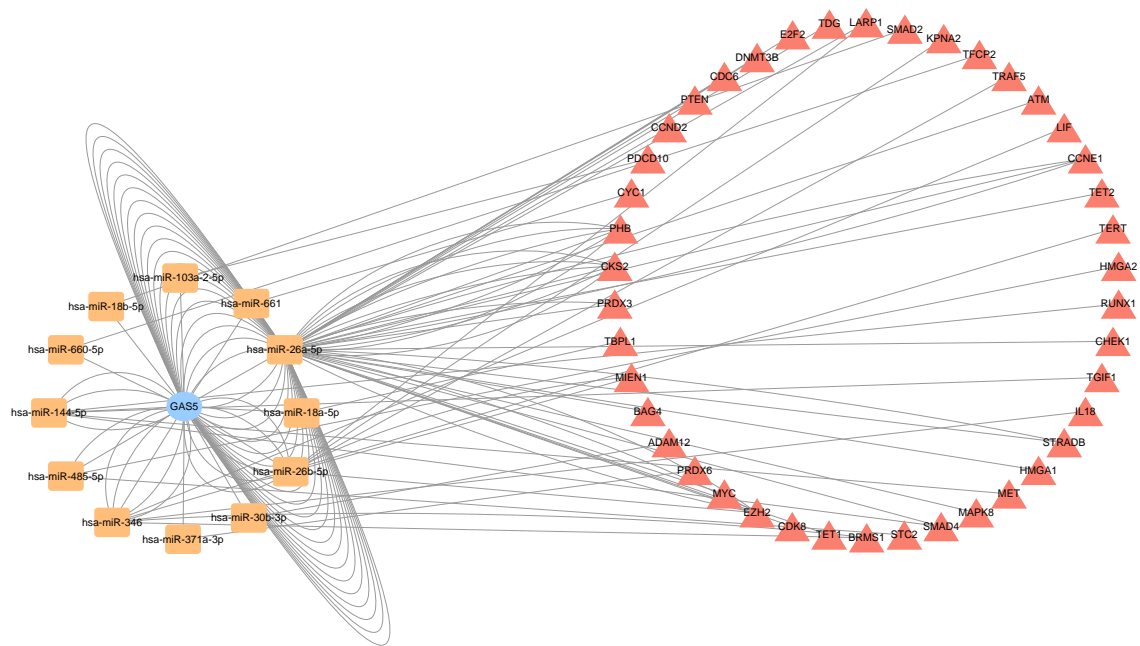

Supplement: Supplementary file 1 [file DataSheet_1.zip › Supplementary Figures/GAS5.pdf]

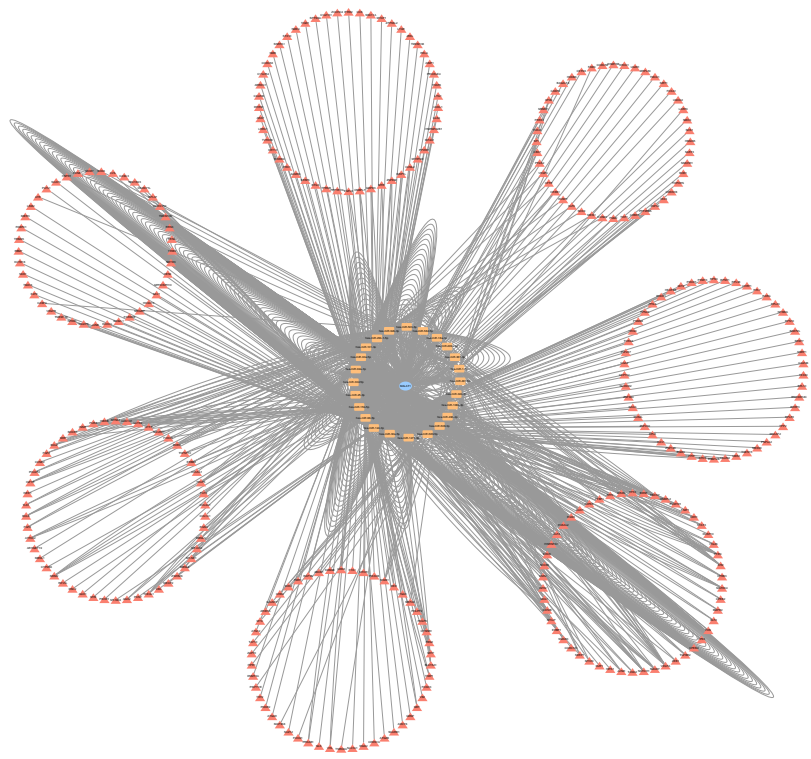

Supplement: Supplementary file 1 [file DataSheet_1.zip › Supplementary Figures/MALAT1.pdf]

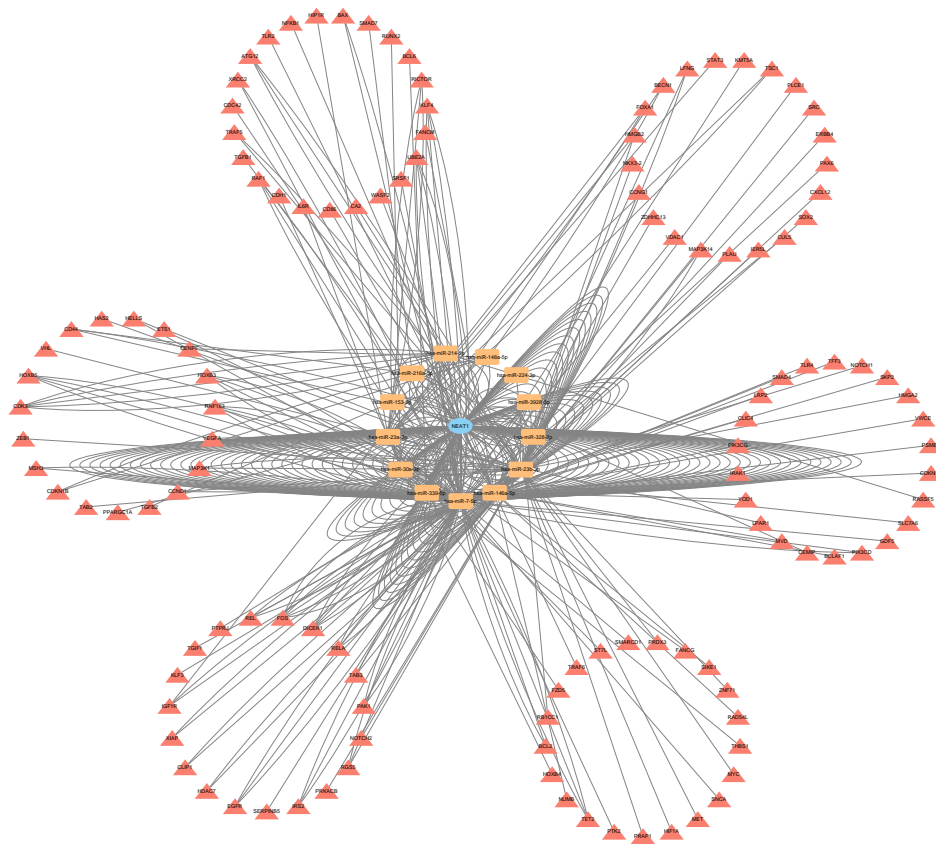

Supplement: Supplementary file 1 [file DataSheet_1.zip › Supplementary Figures/NEAT1.pdf]

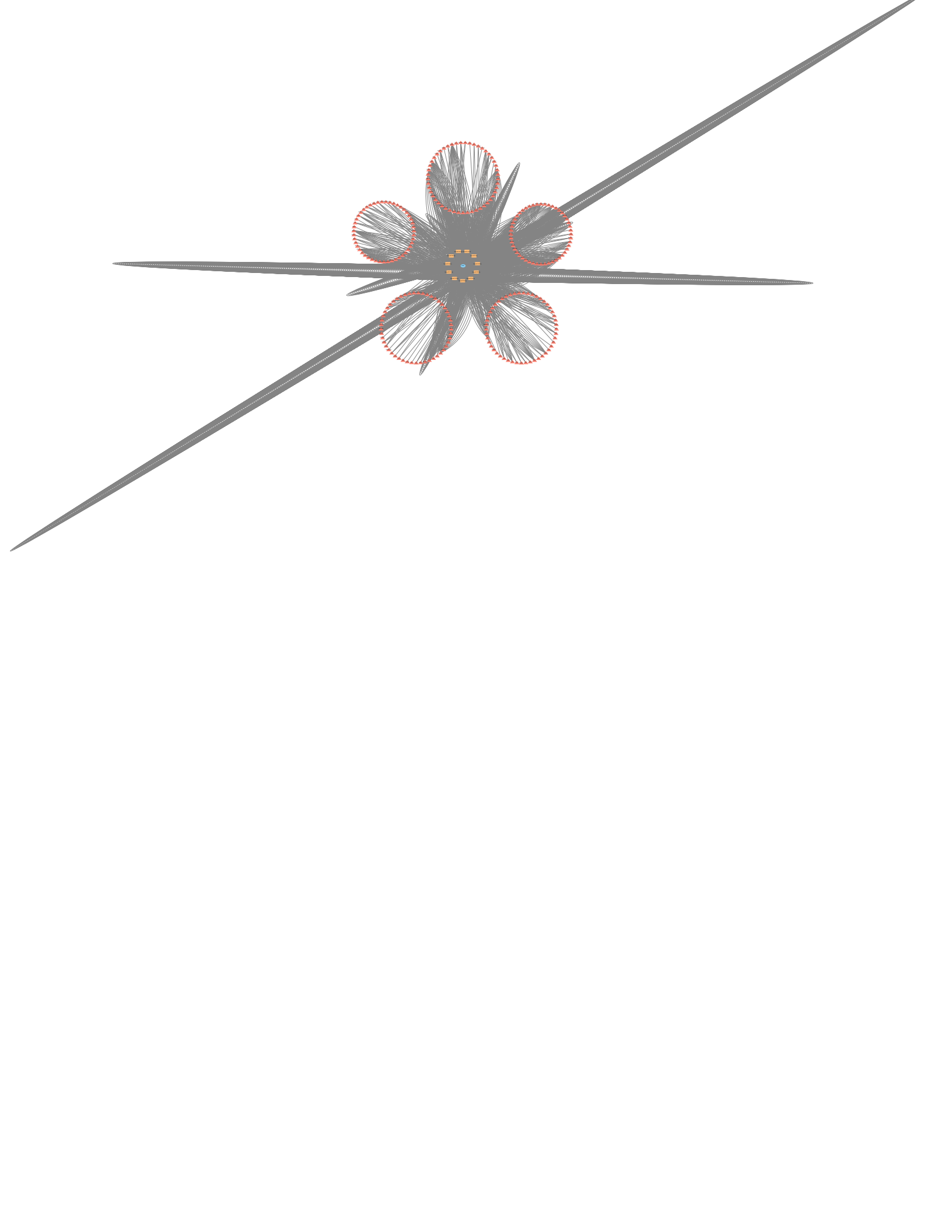

Supplement: Supplementary file 1 [file DataSheet_1.zip › Supplementary Figures/TUG1.pdf]
